# Asymmetric Cluster-Based Measures for Comparative Phylogenetics

**DOI:** 10.1089/cmb.2023.0338

**Published:** 2024-04-22

**Authors:** Sanket Wagle, Alexey Markin, Paweł Górecki, Tavis K. Anderson, Oliver Eulenstein

**Affiliations:** ^1^ Department of Computer Science, Iowa State University, Ames, Iowa, USA.; ^2^ National Animal Disease Center, USDA-ARS, Ames, Iowa, USA.; ^3^ Faculty of Mathematics, Informatics and Mechanics, University of Warsaw, Warsaw, Poland.

**Keywords:** Cluster Affinity, phylogenetic tree, Robinson–Foulds, supertrees

## Abstract

Phylogenetic inference and reconstruction methods generate hypotheses on evolutionary history. Competing inference methods are frequently used, and the evaluation of the generated hypotheses is achieved using tree comparison costs. The Robinson–Foulds (RF) distance is a widely used cost to compare the topology of two trees, but this cost is sensitive to tree error and can overestimate tree differences. To overcome this limitation, a refined version of the RF distance called the Cluster Affinity (CA) distance was introduced. However, CA distances are symmetric and cannot compare different types of trees. These asymmetric comparisons occur when gene trees are compared with species trees, when disparate datasets are integrated into a supertree, or when tree comparison measures are used to infer a phylogenetic network. In this study, we introduce a relaxation of the original Affinity distance to compare heterogeneous trees called the asymmetric CA cost. We also develop a biologically interpretable cost, the Cluster Support cost that normalizes by cluster size across gene trees. The characteristics of these costs are similar to the symmetric CA cost. We describe efficient algorithms, derive the exact diameters, and use these to standardize the cost to be applicable in practice. These costs provide objective, fine-scale, and biologically interpretable values that can assess differences and similarities between phylogenetic trees.

## 1. INTRODUCTION

Phylogenetics is a field of study that investigates the evolutionary relationships between different entities such as genes or species. It involves inferring evolutionary trees or “phylogenies” using computational methods. These trees represent hypotheses about the relationships between different groups, and can be based on data from DNA sequences, protein amino acid sequences, or morphological characters (Yang and Rannala, 2012).

Different datasets and tree inference methods may lead to inconsistent hypotheses of evolutionary relationships. To resolve these inconsistencies, tree comparison costs are used to evaluate the fit between different trees and a given dataset (Allen and Steel, 2001; Estabrook et al., 1985; Robinson and Foulds, 1981; Waterman and Smith, 1978). If the true tree is known, it is possible to compare the different inferred trees and identify those that accurately describe the true tree topology or are close to it (Russo et al., 1996). If the true tree is unknown, which is generally the case in biological studies, cost measures can rank trees and identify areas of incongruence that were generated from different input datasets or tree inference methods (e.g., Shen et al., 2021). Objectively identifying these areas may have utility in phylogenomic studies (Prum et al., 2015; Wickett et al., 2014). Furthermore, one can evaluate the robustness of phylogenetic inference and identify areas of uncertainty by comparing the tree costs associated with different analyses and datasets (Lozano-Fernandez, 2022). Therefore, comparative phylogenetics applies such costs to facilitate phylogenomic studies and provide a metric for assessing tree errors and incongruence.

Tree comparison costs have distinct properties such as their distribution, sensitivity to small changes in the tree structure due to data errors or noise, and computability. Therefore, developing tree comparison costs with beneficial characteristics will enhance research in phylogenetics and other areas that rely upon tree-focused inference. For example, comparison costs are used in epidemiology for comparing transmission trees and virus genealogies (Giardina et al., 2017), to determine horizontal gene transfer events (Bogdanowicz and Giaro, 2017), and in natural language processing to compare and determine an aggregate parse tree (Kulkarni et al., 2022). Consequently, the development and improvement of tree comparison costs is a mature and highly active research area in computational and comparative phylogenetics (e.g., Robinson et al., 2016). The Robinson–Foulds (RF) distance is commonly used to compare the topology of two trees. It measures the cardinality of the symmetric difference of the cluster sets represented by each tree.

Although the RF distance can be computed efficiently, it is highly sensitive to differences in trees, and even small variations can result in a significant RF distance. This property of the RF distance makes it challenging to differentiate between meaningful and random differences, which can lead to incorrect conclusions about the similarity of the compared trees. These issues have been previously discussed by Steel and Penny (1993) and Lin et al. (2011). There have been various approaches proposed in the literature to generalize the RF distance measure. These include matching similar splits between trees (Bogdanowicz and Giaro, 2013; Bogdanowicz and Giaro, 2011), normalizing using the Jaccard Index (Nye et al., 2006), or normalizing through the use of an arbitrary exponent (Böcker et al., 2013). While these methods have improved the application of RF-like distance measures, they do not fully account for the inherent asymmetry in many phylogenetic comparison studies.

Phylogenomic studies often encounter asymmetry issues. For instance, when comparing a gene tree to a species tree, the gene tree may not be fully resolved due to low branch support or insufficient phylogenetic signal in the alignment. As a result, the clusters in the species tree may not be present in the gene tree, which leads to asymmetric comparison costs (Page, 2002; Swenson et al., 2011). Asymmetry in phylogenomics also appears due to gene duplications (i.e., paralogous genes) or horizontal gene transfer, and assessing trees objectively can strengthen phylogenomic studies (Lozano-Fernandez, 2022).

In this work, we introduce the *asymmetric Cluster Affinity* (CA) cost to address the problems of RF. This measure involves determining the minimum cost of a cluster in the source tree for each cluster in the target tree, thereby reducing the issues of RF. We also introduce a biologically interpretable cost related to CA, the Cluster Support (CS) cost that normalizes the individual cluster contributions to the cost by the respective cluster sizes in the source tree. Both costs can be efficiently computed in $O\left(n\log^{3}n\right)$ time for trees of size *n* by adopting the algorithm by Truszkowski et al. (2019). We describe an O(nlogn) time algorithm to study the robustness of our costs against small phylogenetic error. Phylogenetic studies often involve comparing different trees using various costs, which must be normalized to provide meaningful results. To facilitate this normalization, we derive the diameters of our costs, which can be beneficial for practitioners. Our comparative studies have shown that our costs have a broader distribution range and are less skewed than the RF distance. These tree comparison costs can provide objective and biologically interpretable values to evaluate the similarities and differences between phylogenetic trees at a fine scale.

### 1.1. Related work

The RF distance (Robinson and Foulds, 1981) is a standard distance for comparing phylogenies due to its low complexity and interpretability. However, since RF has a narrow distribution and is sensitive to tree errors, there have been many attempts to generalize RF and overcome its shortcomings. A few notable examples are (1) the information-theoretic generalized RF (Smith, 2020) and (2) Matching Split (MS) and Matching Cluster(MC) distances (Bogdanowicz and Giaro, 2013; Bogdanowicz and Giaro, 2011; Lin et al., 2011) that find a minimum perfect matching between the split representation or the cluster representation of two trees. The CA distance later relaxed the matching requirement in the MC distance, thus allowing a one-to-many mapping from one tree to another. This relaxation allowed for a considerably faster distance computation while preserving the robustness to tree error and the wide distribution range that the MC distance has. The CA distance assigns for a pair of clusters *c*_1_ and *c*_2_ a corresponding cost equal to the total number of changes required to change the cluster *c*_1_ into the cluster *c*_2_. The mapping cost for tree *T*_1_ to tree *T*_2_ is the sum of the minimum mapping of all the clusters in *T*_1_ to the clusters in *T*_2_. The CA distance for *T*_1_ and *T*_2_ is the arithmetic mean between the mapping costs from *T*_1_ to *T*_2_ and vice versa.

Another key property of tree comparison measures is the *diameter*, that is, the maximum distance between two trees over a fixed taxon set of size *n*. It is crucial to understand the diameter to make tree distances comparable for different pairs of trees through normalization using the diameter. Additionally, normalization enables comparison across different tree comparison measurements. For RF, the diameter is known, but the exact diameters of MS, MC, and CA remain an open problem (although Bogdanowicz and Giaro, 2013; Bogdanowicz and Giaro, 2011; Moon and Eulenstein, 2019 provide bounds).

### 1.2. Our contribution

In this study, we relax the CA cost, introduce an efficient algorithm for computing updates to the cost in response to Nearest Neighbor Interchange (NNI) tree edit operations, and establish an exact diameter for the cost. We adopt two approaches to relax the cost. First, we allow the cost to be asymmetric by only taking into account the cost of CA mapping from *T*_1_ to *T*_2_. This asymmetry appears naturally when comparing different tree types, such as a gene tree to a species tree in a phylogenomic analysis or an estimated tree to the ground truth in simulation/convergence studies. Second, we permit clusters in *T*_1_ to map to *trivial* clusters in *T*_2_ (i.e., leaves and the root cluster). This was not allowed in the original definition (Moon and Eulenstein, 2019).

Note that the CA cost sums up the raw cluster differences across all clusters. That way, a 5% difference in a bigger cluster with 500 taxa may dominate over a 40% difference in a small cluster with just 10 taxa. To overcome this, we define another measure, where CA is normalized by the respective cluster size, which we denote the *CS* cost. This way all clusters contribute the percentage of the distance rather than the raw numbers as in the original CA cost. A 5% overall CS cost means that clusters in *T*_1_ are, on average, 5% different from clusters in *T*_2_.

We developed an algorithm that efficiently conducts an NNI (Bordewich and Semple, 2005) search with the CA or CS cost. This algorithm requires a single preprocessing step that takes $O\left(n^{2}\right)$ time, and the subsequent NNI iterations only take O(nlogn) time each. Then, Subtree Prune and Regraft (SPR) search can be conducted efficiently using an NNI-graph presented in Chaudhary et al. (2012). As NNI and SPR search strategies are standard for species tree inference heuristics (Bininda-Emonds, 2004), these algorithms make the CA and CS costs directly applicable for phylogenomic inference.

Furthermore, we proved that the CA diameter for trees with *n* leaves is $\left\lceil \frac{n^{2}-2n}{4}\right\rceil$ and the CS diameter is $n-H_{\left\lceil \frac{n}{2}\right\rceil }-H_{\left\lfloor \frac{n}{2}\right\rfloor }$, where *H_i_* is the *i*-th harmonic number. We introduced the concept of a *separation diameter* that measures how asymmetric a cost is; that is, the maximum value of $\left|CA\left(T_{1},T_{2}\right)-CA\left(T_{2},T_{1}\right)\right|$ for all trees *T*_1_ and *T*_2_. We prove that the CA cost is significantly impacted by the topology of *T*_1_, and the separation diameter for CA is in the order of $\Theta \left(n^{2}\right)$. This result implies that, in practice, CA costs need to be normalized by a diameter specific to the topology of *T*_1_. While the exact topology-specific diameter remains an open problem, we provide a practical upper bound on that diameter, supported by our theoretical results. Lastly, we demonstrated that our relaxed CA cost and the novel CS cost are similar to or improve upon the original CA formulation in terms of distribution properties and robustness to error.

## 2. ASYMMETRIC CA COST

A phylogenetic tree *T* over a taxon set *M* is a rooted binary tree where each leaf is bijectively labeled with the elements from *M*. The vertex set and edge set of *T* are denoted by V(T) and E(T), respectively. By L(T) we denote the set of all leaves of *T*, and by the size of *T*, usually denoted as *n*, we define the size of L(T).

An edge (u,v) from E(T) is directed from *u* to *v*, where *v* is a *child* of *u*, and *u* is the *parent* of *v*. For a vertex w∈V(T), Ch(w) is the set of all children of *w*. If two distinct vertices *u* and *v* have the same parent, they are *siblings*. We also define T(v) as the subtree of *T* rooted at *v*.

We define the *height* of a node *v* as the edge-length of the longest path i.e., the path containing the maximum number of edges from the node *v* to a leaf *l* such that l∈L(T(v)). We define the *height* of a tree *T* as the height of the root of *T*.

For two sets *A* and *B*, we define AΔB as the symmetric difference between them. That is, AΔB=(A∖B)∪(B∖A).

A set of leaves L(T(v)) is called a cluster of the node *v* and is denoted by *c_v_*. Note that we identify the leaves in a phylogenetic tree with the respective labels (taxa). A tree *G* can be represented by a set of clusters $C\left(G\right)=\left\{c_{i}|i\in V\left(G\right)\right\}$.

For convenience, we assume throughout the text that *M* is a taxon set, *G*, *S*, and *T* are trees over *M*, and c⊆M is a cluster over *M*.

**Definition 1** (CA cost (Moon and Eulenstein, 2019)). CA cost *from c to S is* $d\left(c,S\right):=\min_{x\in C\left(S\right)}|c\Delta x|$*, and* CA cost *from G to S is* $d\left(G,S\right):=\sum_{c\in C\left(G\right)}d\left(c,S\right)$*, and the* Symmetric CA cost *between G and S is* $d_{sym}\left(G,S\right):=\frac{d\left(G,S\right)+d\left(S,G\right)}{2}$.

**Definition 2** (Diameter of cost function). *The diameter of a cost function between trees is the maximum value that the cost function can have over all trees over the same leaf set.*

### 2.1. Tree edit operations

We define two classic tree edit operations for rooted trees, namely the SPR operation and the NNI operation (Bordewich and Semple, 2005).

Let *T* be a phylogenetic tree and let e=(u,v) be an edge in E(T) and w∈V(T). Then we define the SPR operation SPR(T,v,w)=T′ as the rooted binary tree obtained by deleting *e* and then adjoining edge *f* between *v* and the component *C_u_* that contains *u* in one of the two following ways:

1. If *w* is the root of *T*, we create a new vertex u′ and a new edge from u′ to *w*. Then, we adjoin *f* between u′ and *v* and suppress the degree two vertex *u*. Then, u′ becomes the new root for the tree T′.
2. Otherwise, we create a new vertex, u′ which subdivides the edge whose bottom node is *w* in *C_u_* and adjoining *f* between u′ and *v*. Then, we either suppress the degree-two vertex *u* or if *u* is the root of *T*, delete *u* and the edge incident with *u*, making the other end-vertex of the edge the root.

The *NNI operations* are SPR operations where the subtree is pruned close to its regrafting position as follows. For a nonroot node *v* from *T*, let NNI(T,v) be the SPR operation with the unique edge e=(u,v) from *T* and *w* being the sibling of *u*.

### 2.2. NNI search using CA heuristic

We present an algorithm for the efficient NNI tree space traversal.

**Theorem 1*.*** *Given a cluster c and a tree T*_0_ *of size n*, let $T_{1},T_{2},T_{3},\ldots$ *be a sequence of trees, such that T_i_ is obtained from* $T_{i-1}$ *by a single NNI operation. Then, after* O(n) *preprocessing steps on T*_0_, *one can compute* $d\left(c,T_{i}\right)$ *for each* i>0 *in* O(logn) *time.*

*Proof.* In the previous section, we showed that computing $d\left(c,T_{0}\right)$ can be performed in O(n) time. During this computation, we obtain $\left|c\Delta c_{v}\right|$ values for each $v\in V\left(T_{0}\right)$. For convenience, let $d_{v}:=|c\Delta c_{v}|$. We then place all *d_v_* values in a binary min-heap. Recall that obtaining the minimum value from a binary min-heap can be performed in O(1) time, and changing an element's value can be performed in O(logn) time. Building a min-heap requires O(n) time.

Now, we show how to compute $d\left(c,T_{1}\right)$ and update the min-heap in O(logn) time. Then, computation of $d\left(c,T_{2}\right)$, $d\left(c,T_{3}\right)$, … follows the same algorithm. Let $T_{1}=NNI\left(T_{0},u\right)$, where *u* is not the root of *T*. Let *v* be the parent of *u*, *w* be the sibling of *u*, and *x* be the sibling of *v*. Note that the only cluster that changes after the NNI is *c_v_*. The new cluster in *T*_1_ is $c\prime =c_{u}\cup c_{x}$. Note that we can compute |cΔc′| in constant time as $\left|c\Delta c\prime |=d_{u}+d_{x}-|c\right|$.

Updating *d_v_* in the binary heap by removing the old value and then replacing it with |cΔc′|, can be done in O(logn) time. Then, querying the minimum value from the min-heap will give us $d\left(c,T_{1}\right)$ in constant time.

### 2.3. Diameter of the CA cost

In this section, we derive the diameter for the asymmetric CA cost. However, first we require a few additional definitions to obtain the diameter of the CA cost.

A *rooted caterpillar tree* is a rooted tree *T*, where each internal node has at least one leaf child. We define a caterpillar *C_n_* using the standard nested parenthesis notation as (n,(n−1,…(2,1)…)), where the leaves are numbers. A *perfectly balanced tree* is a rooted tree *T* where each leaf is at the same distance in the number of edges from the root. *The cherry of a caterpillar tree* is the smallest subtree of the caterpillar tree, which contains two leaf nodes. For a tree *T* of the size *n* and a node v∈V(T), we define let $\tau_{T}\left(v\right):=\min\left(n-|c_{v}|,|c_{v}|-1\right)$ and $\tau \left(T\right):=\sum_{v\in V\left(T\right)}\tau \left(v\right)$.

**FIG. 1.**
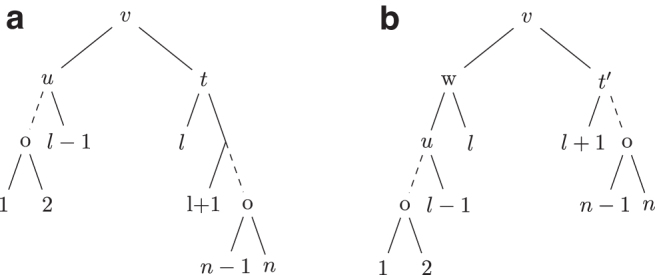
**(a)** Shows a tree *T* where *v* is an internal node with two children *u* and *t* in *T* such that T(u) and T(t) are caterpillar subtrees and |L(T(u))|≥|L(T(t))|≥2. **(b)** Shows the tree T′ obtained after a caterpillar extend operation on *T* where T′=NNI(T,l).

**Definition 3** (Caterpillar-extend). *For a noncaterpillar tree T let v be an internal node in T such that v has two children u and t,* T(u) *and* T(t) *are two caterpillars such that* |L(T(u))|≥|L(T(t))|≥2*. By* T →T′ *we denote the caterpillar-extend operation* T′=NNI(T,l) *on v, where l is a leaf-child of t. See* Figure 1 *for an example of the caterpillar-extend operation.*

**Lemma 1.** *For every node v in a tree T,* $d\left(c_{v},S\right)\leq \tau_{T}\left(v\right)$ *and* d(T,S)≤τ(T) *for all trees S with the same leaf set as T*.

*Proof.* Let *n* be the size of *S*. Consider a leaf *l* on tree *S* such that $l\in c_{v}$. Hence $|c_{v}\Delta \left\{l\right\}|=|c_{v}|-1$. Similarly, for the cluster *M* of the root of the tree *S*, we have $\left|c_{v}\Delta M|=|M|-|c_{v}|=n-|c_{v}\right|$. Since $d\left(c_{v},S\right)=\min_{x\in C\left(S\right)}|c_{v}\Delta x|$ and both clusters {l} and *M* exist in C(S), $d\left(c_{v},S\right)\leq \min\left(\left|c_{v}|-1,n-|c_{v}\right|\right)$. Recall that we have $\tau_{T}\left(v\right)=\min\left(\left|c_{v}|-1,n-|c_{v}\right|\right)$ and $\tau \left(T\right)=\sum_{v\in V\left(T\right)}\left(\tau \left(v\right)\right)$. Hence $d\left(c_{v},S\right)\leq \tau_{T}\left(v\right)$ and ∀T′,d(T,T′)≤τ(T)

**Lemma 2.** If T →T′, then τ(T)≤τ(T′).

*Proof.* Let *n* be the size of *T* and T′. Let *v*, *u*, and *t* be the nodes from the caterpillar-extend operation definition. Note, that $\left|c_{u}|\geq |c_{t}\right|$ and $\tau \left(T\right)=\sum_{i\in V\left(T\right)}\tau_{T}\left(i\right)$.

Let *c_w_* represent the new cluster that is formed in T′. Note that there are only two different clusters between *T* and T′, namely, *c_t_* and *c_w_*. Hence, if $\tau_{T\prime }\left(w\right)\geq \tau_{T}\left(t\right)$ then τ(T)≥τ(T′). Since $\left|c_{u}|\geq |c_{t}\right|$, $|c_{t}|\leq \frac{n}{2}$ and $\tau_{T}\left(t\right)=|c_{t}|-1$. Then, there are two cases for *c_w_*. If $|c_{w}|\leq \frac{n}{2}$, we have $\tau_{T\prime }\left(w\right)=|c_{w}|-1=|c_{u}|>\tau_{T}\left(t\right)$. Otherwise, $|c_{w}|>\frac{n}{2}$ and it follows from $n\geq |c_{t}|+|c_{u}|$ that $\tau_{T}\left(w\right)=n-|c_{w}|=n-|c_{u}|-1\geq \tau_{T}\left(t\right)$. We conclude $\tau_{T\prime }\left(w\right)\geq \tau_{T}\left(t\right)$, which implies τ(T)≥τ(T′).

**Corollary 1.** *Any maximal sequence of caterpillar-extend operations that starts in a tree T terminates in a caterpillar tree* $T^{*}$*. Moreover,* $\tau \left(T^{*}\right)$ *is maximal in the set of all trees of fixed size, and it does not depend on* $T^{*}$*, as long as* $T^{*}$ *is a caterpillar.*

**Lemma 3.** *For any n,* $\tau \left(C_{n}\right)=d\left(C_{n},{\bar{C}}_{n}\right)$ *where* ${\bar{C}}_{n}=\left(1,\left(2,\ldots \left(n,n-1\right)\ldots \right)\right)$.

*Proof.* Consider a cluster *c* from *C_n_*. For any cluster *r* in ${\bar{C}}_{n}$, associated with the vertex $v\in V\left({\bar{C}}_{n}\right)$, such that |c∩r|≥1 and |r|>1, |r|≥n−|c| since there are n−|c| taxa that are above *c* in *C_n_*. More precisely, if |c∩r|≥1 and |r|>1, then |r|=n−|c|+|c∩r|. Thus for a cluster *r* such that |c∩r|≥1 and |r|>1,

|cΔr|=|c|+n−|c|+|c∩r|−2|c∩r|=n−|c∩r|.

Note that |c∩r|=|c| if *r* is the cluster of the root. Also, note that there is always at least one leaf node *l* in ${\bar{C}}_{n}$ such that l∈c and thus |cΔ{l}|=|c|−1. For all the remaining cases, when r∩c=⊘, we have |cΔr|=|c|+|r|. Hence we have,

$$d\left(c,{\bar{C}}_{n}\right)=\min\left(\left|c|-1,n-|c\right|\right)=\tau \left(c\right).$$

Hence,

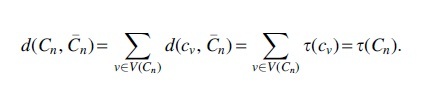

**Theorem 2** (CA cost diameter). *The maximum CA cost between two trees of size n is* $\left\lceil \frac{n^{2}-2n}{4}\right\rceil$.

*Proof.* We show that the diameter is $\tau \left(T^{*}\right)$ where $T^{*}$ is a caterpillar. Let *T* and *S* be two trees over the same set of leaves *M* and let |M|=n. Then, by Lemma 1 d(T,S)≤τ(T). Next, we transform *T* into a caterpillar $T^{*}$ by a sequence of caterpillar-extend operations. By Lemma 2, $\tau \left(T\right)\leq \tau \left(T^{*}\right)$. By Corollary 1, $\tau \left(T^{*}\right)$ is maximal and does not depend on $T^{*}$ as long as $T^{*}$ is a caterpillar. We showed that for any pair of trees, T,S, d(T,S) is bounded by the value $\tau \left(T^{*}\right)$. Since, $\tau \left(T^{*}\right)$ is reached by two caterpillars, by Lemma 3, we conclude that $\tau \left(T^{*}\right)$ is maximal. It remains to derive the exact value:

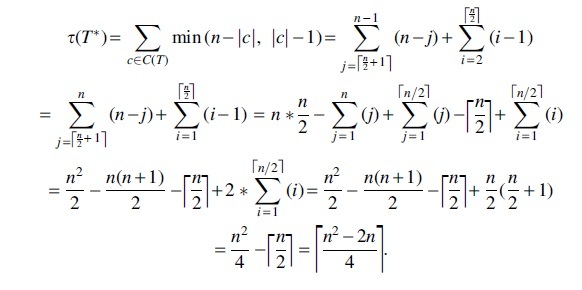

Hence, the diameter for the CA cost is $\left\lceil \frac{n^{2}-2n}{4}\right\rceil .$

### 2.4. Separation diameter

We define the separation cost for CA cost as the absolute difference between the CA costs between the two trees *S* and *G*. That is σ(G,S)=|d(G,S)−d(S,G)|. Similarly, let the *separation diameter* of the CA cost be the diameter of the separation cost for the CA cost. Below we show how to derive a bound for the separation diameter for the CA cost.

**Lemma 4.** *The separation diameter of the CA cost is bound above by* $\left\lceil \frac{n^{2}-2n}{4}\right\rceil$.

*Proof.* It follows from Theorem 2.

**Lemma 5.** *For any* $n=2^{m}$, *there exists a perfectly balanced tree P_n_ such that* $d\left(C_{n},P_{n}\right)=\tau \left(C_{n}\right)=\frac{n^{2}-2n}{4}$.

*Proof.* We construct a perfectly balanced tree *P_n_* such that each nontrivial cluster in *P_n_* is of the form {i,…,j,n−j+1,…,n−i+1} for some $1\leq i\leq j\leq \frac{n}{2}$. We define the labeling in *P_n_* as follows: let the $i^{th}$ cherry in *P_n_* in the prefix order have leaf labels *i* and n−i+1 for 1≤i≤n∕2.

Consider a cluster c={1,2,…,k} in *C_n_* and a nonleaf cluster $p\in P_{n}$. Then p={i,…j,n−j+1,…,n−i+1} for some 1≤i≤j≤n. We have two cases as follows:

1. **Case 1:** if i≤k≤j, then |c∩p|=k−i+1 and thus $|c\cap p|\leq \frac{\left|p\right|}{2}$. Hence, $|c\Delta p|=|c|+|p|-2\left(\left|c\cap p\right|\right)\geq |c|\geq \tau_{G}\left(c\right)$.
2. **Case 2:** if n−j+1≤k≤n−i+1 then |c∩p|=k−(n−j+1)+1+(j−i+1)=k−n+2j−i+1. Thus,

$$\begin{array}{cc} & |c\Delta p|=k+2\left(j-i+1\right)-2\left(k-n+2j-i+1\right)\geq n-k+n-2\left(\frac{n}{2}\right)=n-k=\tau_{G}\left(c\right). \end{array}$$

For all the other remaining cases, while |p| increases, |c∩p| remains constant, and hence they can be ignored. Hence, for every cluster $c\in C\left(C_{n}\right)$, $d\left(c,P_{n}\right)=\tau_{C_{n}}\left(c\right)$. Thus, $d\left(C_{n},P_{n}\right)=\tau \left(C_{n}\right)=\frac{n^{2}-2n}{4}$.

**Lemma 6.** *For a perfectly balanced tree T of size* $n=2^{m}$, τ(T)=nlogn−3n+2.

*Proof.* Let *v* be an internal node in V(T) such that *v* is not the root of *T* and *v* has height *h*. Hence, $|c_{v}|=2^{h}$. Since *T* is a perfectly balanced tree $|c_{v}|\leq \frac{n}{2}$ and thus $\tau \left(c_{v}\right)=|c_{v}|-1$. Thus, the total contribution of nodes with height *h* to τ(T) is $\left(2^{h}-1\right).2^{m-h}$. Hence,

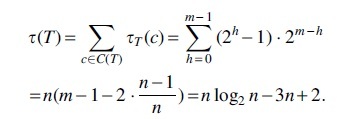

Thus τ(T)=nlogn−3n+2.

**Lemma 7.** *For every n, there exists a caterpillar tree G and a tree S such that the separation diameter* $\sigma \left(G,S\right)\geq \left\lceil \frac{n^{2}-4n}{16}\right\rceil -n\log_{2}n-3n+2$.

*Proof.* It follows from Lemma 6 and Lemma 1 that 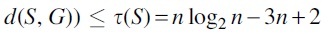 and hence, $\sigma \left(G,S\right)\geq \left\lceil \frac{n^{2}-2n}{4}\right\rceil -\left(n\log_{2}n-3n+2\right)$ when $n=2^{m}$ for some *m*.

For the remaining n′s, we prove it by construction. Let $S_{n}=\left(n,\left(n-1,\left(n-2,\ldots \left(n-n\prime +1,P_{n\prime }\right)\ldots \right)\right)\right)$ where $n\prime =2^{\left\lfloor \log_{2}n\right\rfloor }$ and $P_{n\prime }$ is a perfectly balanced tree of the size n′ from the proof of Lemma 6. Since all clusters of size larger than n′ are present in *C_n_* and $n>n\prime \geq \frac{n}{2}$, we have $\sigma \left(C_{n},S_{n}\right)\geq \sigma \left(C_{n\prime },P_{n\prime }\right)\geq \left\lceil \frac{{\left(n\prime \right)}^{2}-2n\prime }{4}\right\rceil -n\prime \log_{2}n\prime -3n\prime +2\geq \left\lceil \frac{n^{2}-4n}{16}\right\rceil -n\log_{2}n-3n+2.$

**Theorem 3.** *The separation diameter is* $\Theta \left(n^{2}\right)$.

*Proof.* From Lemma 7, $\sigma \left(G,S\right)\geq \left\lceil \frac{n^{2}-4n}{16}\right\rceil -n\log_{2}n-3n+2$ and hence $\sigma \left(G,S\right)=\Omega \left(n^{2}\right)$. Since the CA distance is bound by $O\left(n^{2}\right)$, the separations affinity distance is also $O\left(n^{2}\right)$. Hence, the separation diameter is $\Theta \left(n^{2}\right)$.

### 2.5. CS cost

We define the CS cost and then derive the exact diameter for that cost.

**Definition 4** (CS cost). *The* CS cost *from cluster c to tree S is* $d\prime \left(c,S\right):=\min_{x\in C\left(S\right)}\frac{\left|c\Delta x\right|}{\left|c\right|}$ *and the CS cost from tree G to tree S is* $d\prime \left(G,S\right):=\sum_{c\in C\left(G\right)}d\prime \left(c,S\right)$.

For interpretability, in practice, we further divide d′(G,S) by |C(G)|. Note that the CS cost can be computed in the same way as the CA in $O\left(n\log^{3}n\right)$ time, where *n* is the size of a tree. We further introduce some tree operations to derive the diameter for the CS cost.

**Definition 5** (Caterpillar swap). *For a tree T, let v be an internal node in T such that* T(v) *is a caterpillar tree and u is the sibling of v with two caterpillar subtrees* T(x) *and* T(y) *such that* $\left|c_{v}|<|c_{y}|<|c_{u}\right|$ *and* $\left|c_{y}|\leq |c_{x}\right|$*. Let* T′=NNI(T,x)*. By the caterpillar-swap on x, we call the transformation of T into* T′, *which is denoted by* $T\to^{swap}T\prime$.

**Definition 6** (Caterpillar split). *For a tree T, let v be an internal node in T such that* T(v) *is a caterpillar tree and u is the sibling of v with two caterpillar subtrees* T(x) *and* T(y) *such that* $\left|c_{y}|\leq |c_{v}\right|$ *and* $\left|c_{y}|\leq |c_{x}\right|$*. Let* T′=SPR(T,l,v) *where l is the leaf-child of y. By the caterpillar-split on y, we call the transformation of T into* T′, *which is denoted by* $T\to^{split}T\prime$.

**Definition 7** (Caterpillar-balance). *For a tree T, let* T(u) *and* T(v) *be two caterpillars subtrees of the root such that* $|c_{v}|<|c_{u}|-1$*. By* T⇀T′ *we denote the caterpillar-balance operation* T′=NNI(T,l)*, where l is a leaf-child of u*.

For a tree *T* with a leaf set *M* and a node v∈V(T) let $\phi_{T}\left(v\right):=\frac{\tau_{T}\left(v\right)}{\left|c_{v}\right|}$ and $\phi \left(T\right):=\sum_{v\in V\left(T\right)}\phi_{T}\left(v\right)$.

**Lemma 8.** *For every node v in a tree T*, $d\prime \left(c_{v},S\right)\leq \phi_{T}\left(v\right)$ *and for every tree S*, d′(T,S)≤ϕ(T).

*Proof.* We know from Lemma 1 that $d\left(c_{v},S\right)\leq \tau_{T}\left(v\right)$. Hence, $d\prime \left(c_{v},S\right)\leq \phi_{T}\left(v\right)$. Consequently, we have d′(T,S)≤ϕ(T).

We define a 2-caterpillar tree *D_n_* such that the two subtrees from the root are $C_{\left\lceil \frac{n}{2}\right\rceil }$ and $C_{\left\lfloor \frac{n}{2}\right\rfloor }$. To derive the diameter of the CS cost, we show that there exists a sequence of operations, see Rules 1–5 below that can transform any tree *T* into a 2-caterpillar tree and that $\phi \left(D_{n}\right)$ is maximal over all trees of size *n*.

Let *T* be a tree of the size *n*. Let u,v be siblings in V(T) and let x,y be children of *u*. Assume without loss of generality that $\left|c_{y}|\leq |c_{x}\right|$ and $\left|c_{v}|\leq |c_{u}\right|$. We define the following rules to transform *T* into a 2-caterpillar tree.

Rule 1. If $|c_{v}|\leq \left\lceil \frac{n}{2}\right\rceil$ and T(v) is a noncaterpillar tree, transform *T* by applying caterpillar extend operations on *v* until the subtree rooted at *v* is not a caterpillar.

Rule 2. If T(x) and T(y) are caterpillar subtrees such that $|c_{x}|<\left\lceil \frac{n}{2}\right\rceil$ and $|c_{u}|>\left\lceil \frac{n}{2}\right\rceil$, perform the caterpillar extend operation on *u* (provided the operation is allowed) repeatedly until one of the child subtrees of *u* has exactly $\left\lceil \frac{n}{2}\right\rceil$ leaves.

Rule 3. If T(v),T(x),T(y) are caterpillar subtrees and T(u) is a noncaterpillar subtree such that $\left|c_{v}|<|c_{y}\right|$ and $|c_{x}|\geq \left\lceil \frac{n}{2}\right\rceil$, then perform the caterpillar swap operation on *x*.

Rule 4. If T(v),T(x),T(y) are caterpillar subtrees and T(u) is a noncaterpillar subtree such that $\left|c_{y}|\leq |c_{v}\right|$ and $|c_{x}|\geq \left\lceil \frac{n}{2}\right\rceil$, then perform the caterpillar split operations on a child of *u* repeatedly until such a transformation is not allowed. Note that after the application of Rule 4, one child of *u* becomes a leaf.

Rule 5. If *u* and *v* are children of the root such that T(u) and T(v) are caterpillar trees and $|c_{v}|<|c_{u}|-1$, then perform the caterpillar balance operation.

We show in the following lemma that applying these rules to a tree *T* to obtain a tree T′ always results in an increase in the value of ϕ.

**Lemma 9.** *For a tree* T′ *obtained by applying a rule to a tree T*, ϕ(T′)>ϕ(T).

*Proof.* Let *n* be the size of *T* and T′. Since each rule consists of caterpillar-extend, caterpillar-balance, caterpillar-swap, and caterpillar-split operations, it suffices to prove that for *S* obtained by applying an operation to *T*, ϕ(S)>ϕ(T).

T →S**:** Note that *T* and *S* only differ by two clusters. Let $c_{t},c_{w}$ be the differing clusters as defined in the caterpillar-extend operation. Note that by the definitions of Rule 1 and Rule 2, $|c_{t}|<|c_{w}|\leq \left\lceil \frac{n}{2}\right\rceil$, which implies $\tau_{S}\left(w\right)=|c_{w}|-1$ and $\tau_{T}\left(t\right)=|c_{t}|-1$. Hence, $\phi_{S}\left(w\right)=\frac{|c_{w}|-1}{\left|c_{w}\right|}=1-\frac{1}{\left|c_{w}\right|}>1-\frac{1}{\left|c_{t}\right|}=\phi_{T}\left(t\right)$.

**FIG. 2.**
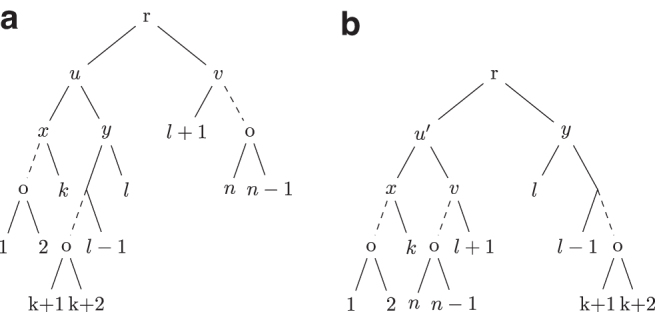
**(a)** Shows a tree *T* where u,v are siblings such that T(v) is a caterpillar tree *u* has two caterpillar subtrees T(x) and T(y) such that |L(T(y))|≤|L(T(x))| and |L(T(v))|<|L(T(y))|. **(b)** Shows the tree T′ obtained after a caterpillar swap operation on *T* where T′=NNI(T,x).

$T\to^{swap}S$**:** Using the notation from Figure 2, note that only *u* and u′ change between *T* and *S*. Moreover, $|c_{u}|>|c_{u\prime }|>\left\lceil \frac{n}{2}\right\rceil$ since $|c_{x}|\geq \left\lceil \frac{n}{2}\right\rceil$. Hence, $\phi_{S}\left(u\prime \right)=\frac{n-|c_{u\prime }|}{\left|c_{u\prime }\right|}>\frac{n-|c_{u}|}{\left|c_{u}\right|}=\phi_{T}\left(u\right)$.

**FIG. 3.**
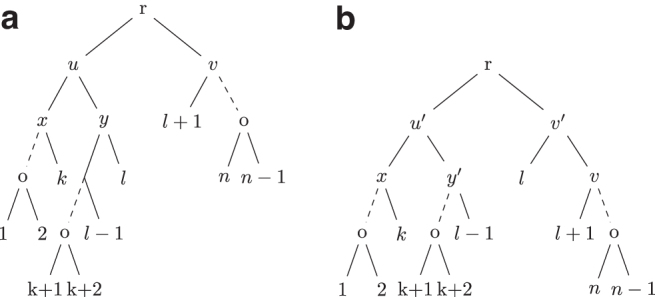
**(a)** Shows a tree *T* where u,v are siblings such that T(v) is a caterpillar tree and *u* has two caterpillar subtrees T(x) and T(y) such that |L(T(y))|≤|L(T(v))| and |L(T(y))|≤|L(T(x))|. **(b)** Shows the tree T′ obtained after a caterpillar-split operation on *T* where T′=SPR(T′,l−1,v).

$T\to^{split}S$**:** Using the notation from Figure 3, *T* and *S* differ by four clusters $c_{u},c_{y},c_{v\prime },c_{u\prime }$. Note that $|c_{u}|>|c_{u\prime }|>\left\lceil \frac{n}{2}\right\rceil$ since $|c_{x}|\geq \left\lceil \frac{n}{2}\right\rceil$. Moreover, $|c_{y}|<|c_{v\prime }|<\left\lceil \frac{n}{2}\right\rceil$. Hence, $\phi_{T_{i}}\left(u\prime \right)=\frac{n-|c_{u\prime }|}{\left|c_{u\prime }\right|}>\frac{n-|c_{u}|}{\left|c_{u}\right|}=\phi_{T}\left(u\right)$ and $\phi_{S}\left(v\prime \right)=\frac{|c_{v\prime }|-1}{\left|c_{v\prime }\right|}=1-\frac{1}{\left|c_{v\prime }\right|}>1-\frac{1}{\left|c_{y}\right|}=\phi_{T}\left(y\right)$.

**FIG. 4.**
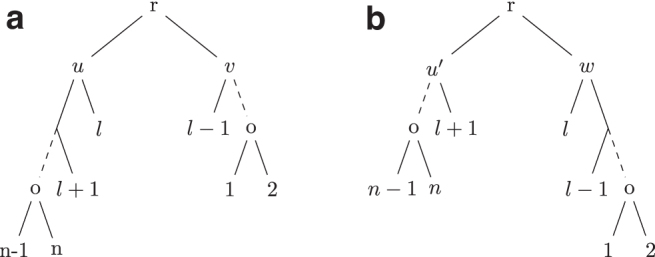
**(a)** Shows a tree *T* where u,v are children of the root of *T* such that T(u) and T(v) are caterpillar subtrees and |L(T(v))|<|L(T(u))|−1. **(b)** Shows the tree T′ obtained after a caterpillar-balance operation on *T* where T′=NNI(T,l).

T⇀S**:** Note that *T* and *S* differ by only two clusters. Let *c_u_* and *c_w_* be the two clusters as defined in the caterpillar-balance operation from Figure 4. Hence $|c_{w}|=|c_{v}|+1$, which implies $n=|c_{u}|+|c_{w}|-1$. Moreover, as $|c_{v}|<\left\lceil \frac{n}{2}\right\rceil$, $\left|c_{w}|\leq \left\lceil \frac{n}{2}\right\rceil <|c_{u}\right|$. Hence, we have $\phi_{S}\left(w\right)=\frac{|c_{w}|-1}{\left|c_{w}\right|}=\frac{n-|c_{u}|}{\left|c_{w}\right|}>\frac{n-|c_{u}|}{\left|c_{u}\right|}=\phi_{T}\left(u\right)$.

Hence, if *S* is obtained by applying an operation to *T*, ϕ(S)>ϕ(T), which implies ϕ(T′)>ϕ(T) where T′ is obtained by applying a rule to *T*.

Hence, for any tree T′ obtained from a tree *T* through an application of one of rules, ϕ(T′)>ϕ(T). We further prove that for any tree *T*, we can apply a rule to *T* if and only if *T* is not a 2-caterpillar tree.

**Lemma 10.** *T is not a* 2-*caterpillar tree if and only if there is a rule that can be applied to T*.

*Proof.* Let *n* be the size of *T*.

(⇒) If *T* is a caterpillar tree such that *w* and *t* are the children of the root and $\left|c_{w}|<|c_{t}\right|$ and *T* has at least three leaves then we can apply Rule 5 to *T* as $|c_{w}|=1$ and $|c_{t}|=n-1$. Hence, *T* cannot be a caterpillar tree. Let *u* be a node in *T* such that T(u) is not a caterpillar tree and *u* has two children, *x* and *y* such that T(x) and T(y) are caterpillar trees. Assume without loss of generality that $\left|c_{y}|\leq |c_{x}\right|$. If *u* is the root of *T*, then we have $|c_{y}|+|c_{x}|=n$, which implies $|c_{y}|<|c_{x}|-1$ since *T* is not a 2-caterpillar tree and Rule 5 can be applied.

Hence, *u* must be a nonroot internal node. If $|c_{u}|\leq \left\lceil \frac{n}{2}\right\rceil$ then Rule 1 can be applied to *T*. Hence $|c_{u}|>\left\lceil \frac{n}{2}\right\rceil$. Furthermore, $|c_{x}|\geq \left\lceil \frac{n}{2}\right\rceil$ since otherwise Rule 2 can be applied to *T*. Let *v* be the sibling of *u*. Note that T(v) must be a caterpillar tree, since $|c_{v}|\leq \left\lceil \frac{n}{2}\right\rceil$ and Rule 1 could be applied to *T* otherwise. Hence, if $\left|c_{v}|<|c_{y}\right|$, Rule 3 can be applied to *T* and conversely if $\left|c_{v}|\geq |c_{y}\right|$, Rule 4 can be applied to *T*.

(⇐) Assume conversely that *T* is a 2-caterpillar tree. Let *u* be an internal node in *T* and let *x* and *y* be the children of *u*. Assume without loss of generality that $\left|c_{y}|\leq |c_{x}\right|$. If *u* is the root node, $|c_{y}|\geq |c_{x}|-1$. Conversely, if *u* is not the root node, then T(u) is a caterpillar tree. Hence, there is no rule that can be applied to *T* if *T* is a 2-caterpillar tree.

Hence, for any non 2-caterpillar tree *T*, there exists a rule that can be applied to *T*. Conversely, if *T* is a 2-caterpillar tree, there is no rule that can be applied to *T*. We showed by Lemma 9 that if we derive a tree T′ by applying a rule to a tree *T*, then ϕ(T′)>ϕ(T). This allows us to repeatedly apply the rules to derive the maximum value of ϕ in the set of all trees of fixed size.

We say that a tree *T* is transformed into T′ by a sequence of rule applications if there is a sequence of trees $T=T_{0},T_{1},\ldots ,T_{l}=T\prime$ with l≥0 such that $T_{i+1}$ is obtained from *T_i_* by an application of a rule. We show that for any tree *T*, there is a maximal sequence of rule applications that can transform *T* into a 2-caterpillar tree T′.

**Lemma 11.** *Every maximal sequence of rule applications is finite and terminates with a 2-caterpillar tree.*

*Proof.* By Lemma 10, for any tree *T* of size *n*, if *T* is not a 2-caterpillar tree then there exists a rule that can be applied to *T*. Moreover, by Lemma 9, for a tree T′ derived by an application of a rule to *T*, ϕ(T)<ϕ(T′). Note that for any tree *T*, ϕ(T)≤2n−1 since for any node v∈V(T), $\phi_{T}\left(v\right)\leq 1$. We first show that for *S* obtained by applying an operation to *T*, $\phi \left(S\right)-\phi \left(T\right)>\frac{1}{n^{2}}>0$. We use the same notation as described in Lemma 9.

T →S: For *c_w_* and *c_t_*, we have $\phi_{S}\left(w\right)-\phi_{T}\left(t\right)=1-\frac{1}{\left|c_{w}\right|}-1+\frac{1}{\left|c_{t}\right|}=\frac{1}{\left|c_{t}\right|}-\frac{1}{\left|c_{w}\right|}=\frac{\left|c_{w}|-|c_{t}\right|}{\left|c_{w}|.|c_{t}\right|}>\frac{1}{n^{2}}$.

$T\to^{swap}S:$ For *c_u_* and $c_{u\prime }$, we have $\phi_{S}\left(u\prime \right)-\phi_{T}\left(u\right)=\frac{n-|c_{u\prime }|}{\left|c_{u\prime }\right|}-\frac{n-|c_{u}|}{\left|c_{u}\right|}=n\cdot \left(\frac{1}{\left|c_{u\prime }\right|}-\frac{1}{\left|c_{u}\right|}\right)=n\cdot \frac{\left|c_{u}|-|c_{u\prime }\right|}{\left|c_{u}|.|c_{u\prime }\right|}>\frac{1}{n^{2}}$.

$T\to^{split}S:$ For *c_u_* and $c_{u\prime }$, we have the same result as above. Similarly for $c_{v\prime }$ and *c_y_*, we have $\phi_{S}\left(v\prime \right)-\phi_{T}\left(y\right)=\frac{1}{\left|c_{y}\right|}-\frac{1}{\left|c_{v\prime }\right|}=\frac{\left|c_{v\prime }|-|c_{y}\right|}{\left|c_{y}|.|c_{v\prime }\right|}>\frac{1}{n^{2}}$.

T⇀S: For $\left|c_{u}\right|$ and $\left|c_{w}\right|$, we have $\phi_{S}\left(w\right)-\phi_{T}\left(u\right)=\frac{|c_{w}|-1}{\left|c_{w}\right|}-\frac{n-|c_{u}|}{\left|c_{u}\right|}=\frac{n-|c_{u}|}{\left|c_{w}\right|}-\frac{n-|c_{u}|}{\left|c_{u}\right|}=\left(n-|c_{u}|\right)\cdot \left(\frac{\left|c_{u}|-|c_{w}\right|}{\left|c_{u}|.|c_{w}\right|}\right)>\frac{1}{n^{2}}$.

We proved that every rule application increases ϕ(T) by at least $\frac{1}{n^{2}}$. Thus, every sequence of rule applications has at most $\left(2n-1\right)\cdot n^{2}$ elements. We conclude that the sequence is finite and must terminate with a 2-caterpillar by Lemma 10.

**Corollary 2.** $\phi \left(T^{*}\right)$ *is maximal in the set of all trees of fixed size and it does not depend on* $T^{*}$, *as long as* $T^{*}$ *is a 2-caterpillar.*

Hence, we have for every tree *T*, a maximal finite sequence of transformations *S_T_* that can transform a tree *T* into a 2-caterpillar tree $T^{*}$. Note that since the transformations are defined by the rules above, we can show that $\phi \left(T^{*}\right)>\phi \left(T\right)$. It remains to show that there exists a scenario that would lead to $\phi \left(T^{*}\right)$. We show that for any 2-caterpillar tree $T^{*}$, we can construct such a tree ${\bar{T}}^{*}$ where $d\prime \left(T^{*},{\bar{T}}^{*}\right)=\phi \left(T^{*}\right)$.

**Lemma 12.** *For any n*, $\phi \left(D_{n}\right)=d\prime \left(D_{n},{\bar{D}}_{n}\right)$ *where* ${\bar{D}}_{n}$ *is a 2-caterpillar tree such that the subtrees of the root are* ${\bar{C}}_{\left\lceil \frac{n}{2}\right\rceil }$ *and* ${\bar{C}}_{\left\lfloor \frac{n}{2}\right\rfloor }$.

*Proof.* Let u,w be the two children of the root of *D_n_*. For *D_n_*, $\phi \left(D_{n}\right)=\sum_{v\in V\left(D_{n}\right)}\phi_{D_{n}}\left(v\right)=\sum_{v\in V\left(D_{n}\left(u\right)\right)}\phi_{D_{n}}\left(v\right)+\sum_{v\in V\left(D_{n}\left(w\right)\right)}\phi_{D_{n}}\left(v\right)+\phi_{D_{n}}\left(r\right)$ where *r* is the root node of *D_n_*. Note $\phi_{D_{n}}\left(r\right)=0$. By Lemma 3, for every node $v\in V\left(D_{n}\left(u\right)\right)$, $d\left(c_{v},{\bar{D}}_{n}\right)=\tau_{D_{n}}\left(v\right)$, which implies $d\prime \left(c_{v},{\bar{D}}_{n}\right)=\phi_{D_{n}}\left(v\right)$. Similarly, for every node $v\in V\left(D_{n}\left(w\right)\right)$, $d\left(c_{v},{\bar{D}}_{n}\right)=\tau_{D_{n}}\left(v\right)$, which implies $d\prime \left(c_{v},{\bar{D}}_{n}\right)=\phi_{D_{n}}\left(v\right)$. Hence,

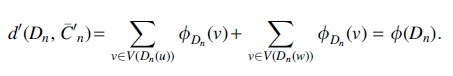


Hence, for any tree *T* we are able to convert it into a 2-caterpillar tree $T^{*}$ by the sequence of operations defined in Lemma 11. Moreover, by Lemma 9 we know that $\phi \left(T^{*}\right)>\phi \left(T\right)$ and by Lemma 12 we can construct a tree T′ such that $d\prime \left(T^{*},T\prime \right)=\phi \left(T^{*}\right)$. Thus, as $\phi \left(T^{*}\right)$ is the maximal value of ϕ, we derive the diameter for the CS cost as the value of $\phi \left(T^{*}\right)$.

**Theorem 4** (CA support cost diameter). *The maximum CS cost between two trees of size n is* $n-H_{\left\lceil \frac{n}{2}\right\rceil }-H_{\left\lfloor \frac{n}{2}\right\rfloor }$, *where H_i_ is the i*-*th harmonic number.*

*Proof.* We show that the diameter is $\phi \left(T^{*}\right)$ where $T^{*}$ is a 2-caterpillar. Let *T* and *S* be two trees over the same set of leaves *M* and let |M|=n. Then, by Lemma 8, d′(T,S)≤ϕ(T). Next, we transform *T* into a 2-caterpillar tree $T^{*}$ by the sequence described in Lemma 11. By Corollary 2, we showed that for any pair of trees, T,S, d(T,S) is bounded by the value $\phi \left(T^{*}\right)$. Since, $\phi \left(T^{*}\right)$ is reached by two 2-caterpillar trees by Lemma 12, we conclude that $\phi \left(T^{*}\right)$ is maximal. It remains to derive the exact value. Let u,w be the children of the root of $T^{*}$.

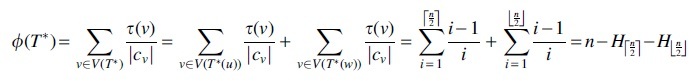

Hence the diameter for the CS cost is $n-H_{\left\lceil \frac{n}{2}\right\rceil }-H_{\left\lfloor \frac{n}{2}\right\rfloor }$.

## 3. EMPIRICAL STUDY

We compare the distribution and the robustness of the CA and CS costs (defined in this work) with the classic RF distance. Since the CA and CS are asymmetric costs, we use the asymmetric (one-sided) version of RF for comparison, also known as the false negative rate. We define the one-sided RF between trees *T*_1_ and *T*_2_ as the number of clusters in *T*_1_ that are not present in *T*_2_.

### 3.1. CA and CS display a broad distribution range

Table 1 shows the descriptive statistics of the (asymmetric) CA, CS, and RF costs on pairs of random trees. We generated 10,000 pairs of trees each containing 100 taxa. We used the birth–death model to generate the trees with a birth rate of 1.0 and a death rate of 0.5. The birth–death process was terminated when the tree had the required number of extant taxa.

**Table 1.** Asymmetric Robinson–Foulds, Cluster Affinity, and Cluster Support Distribution Statistics for Random Tree Pairs with n∈{100,1000} Leaves

Leaves		RF	CA	CS	
100	Mean	0.997	0.816	0.958	
	SD	0.004	0.056	0.017	
	Median	1.000	0.810	0.961	
	Min	0.969	0.687	0.865	
1000	Mean	0.999	0.883	0.993	
	SD	$4.857\times 10^{-4}$	0.036	0.002	
	Median	1.000	0.878	0.994	
	Min	0.996	0.807	0.982	

All values are normalized by the maximum observed cost, respectively. Note that both CA and CS display a broader distribution range than RF. Moreover, out of all the costs, CA is the least skewed toward the maximum while RF is the most skewed toward the maximum.

CA, Cluster Affinity; CS, Cluster Support; RF, Robinson–Foulds.

**FIG. 5.**
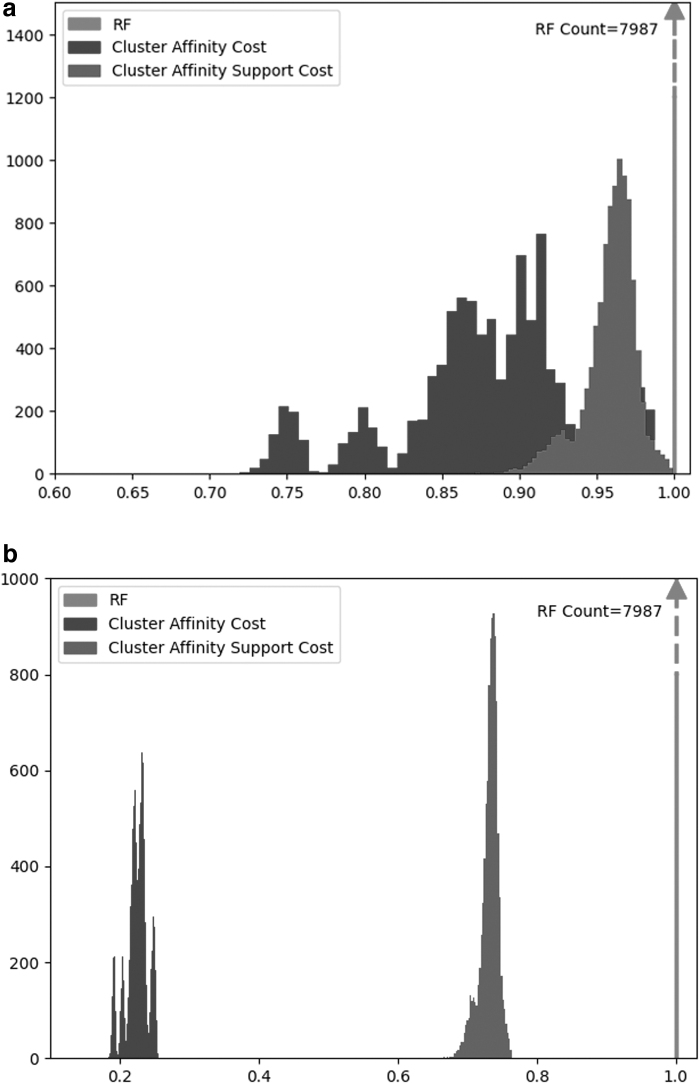
**(a, b)** Show the distribution of the three costs when normalized by the observed maximum or the theoretical diameter, respectively. Note that both the CA cost and the CS cost have a broader distribution range than the RF cost. Furthermore, under RF, 80% of tree pairs had a cost of 1.0 (the theoretical maximum). **(a)** RF, CA, and CS costs for repeated NNI operations normalized by the observed maxima. **(b)** RF, CA, and CS costs for repeated SPR operations normalized by the observed maxima. CA, Cluster Affinity; CS, Cluster Support; NNI, Nearest Neighbor Interchange; RF, Robinson–Foulds; SPR, Subtree Prune and Regraft.

We observe that CA and CS both have a broader distribution range (standard deviation and the min–max range) than RF. Out of all three costs, CA has the broadest range and has the least skewed distribution, while RF is most skewed toward the maximum. These results are similar to the comparison between the original (symmetric) CA distance and RF (Moon and Eulenstein, 2019); thus, demonstrating that our cost relaxation maintained the key properties of the original CA distance. Figure 5 visually captures the distributions for CA and CS. Note that we truncate the data for RF to preserve the details of the histograms for CA and CS. Moreover, due to the large diameter of the CA cost, the discretization of the bins on the histogram results in multiple peaks being observed.

Additionally, we quantified the CA asymmetry between the tree pairs. For a pair of trees ($T_{1},T_{2}$) we computed the separation cost $\left|d\left(T_{1},T_{2}\right)-d\left(T_{2},T_{1}\right)\right|$ and normalized by the maximum observed CA cost in our dataset. The average separation cost was 0.057 with a maximum value of 0.270 and a standard deviation of 0.041. That is, the asymmetry between birth–death trees was 6% on average and 27% in the worst case.

We observed that for a pair of trees with n∈{100,1000}, it required an average of 1.5 ms and 17 ms to compute the asymmetric RF distance, while it required an average of 123 ms and 13,595 ms to compute the CA cost. This is due to the higher computational complexity and resolution of the CA cost. In this study, we used a quadratic algorithm for computing the CA and CS costs. This algorithm computes the symmetric difference between each pair of clusters in the two trees and is required for the NNI algorithm from Section 2.2. The experiments were run on Ubuntu 22.04 with an Intel i7 CPU and 64GB of RAM with Python 3.10 and Dendropy 4.5.2.

### 3.2. CA and CS are robust to tree edit operations

We demonstrate that CA and CS are significantly more robust to tree edit operations (and, hence, tree-error) than RF. Our experimental setup follows (Moon and Eulenstein, 2019) for comparison between the CA, CS, and RF distances.

#### 3.2.1. Dataset

We generated a set of random trees $T_{1},T_{2},\ldots ,T_{100}$ where each tree had 100 leaves using the birth–death model with birth rate 1.0 and death rate 0.5. For each tree *T_i_*, we generated a sequence of trees 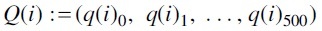, where each 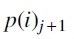 was obtained by an NNI operation on 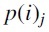 and the edge for the NNI operation was chosen uniformly and independently at random. Similarly, for each tree *T_i_*, we also generated a sequence of trees 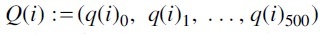 where each 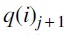 was obtained by an SPR operation on 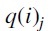 where the edges for the SPR operation were chosen uniformly and independently at random. Note that 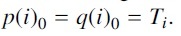


#### 3.2.2. Experimental setting

Distances were computed between tree pairs 
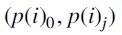
 and 
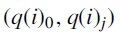
 for each *j* and averaged over all i∈{1,…,100} under the RF cost, CA cost, and CS cost.

#### 3.2.3. Results

**FIG. 6.**
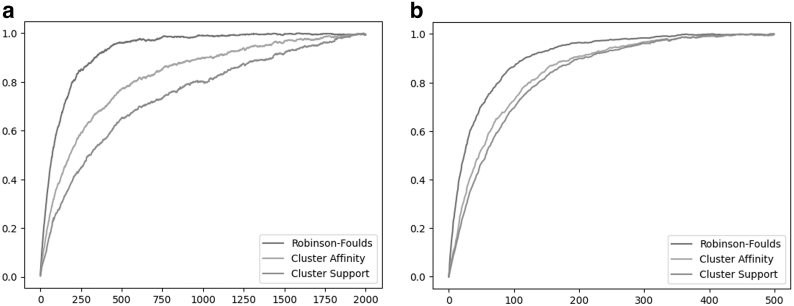
**(a, b)** Show the changes in RF, CA, and CS costs for subsequent NNI and SPR operations, respectively. Note that the RF distance approaches the maximum value more rapidly than the CA and CS costs.

The respective RF, CA, and CS costs over consecutive NNI and SPR operations are shown in Figure 6. We observe that for both NNI and SPR edit operations, RF approaches the maximum value very rapidly. In contrast, CA and CS costs are significantly more robust to the tree edits and demonstrated better resolution than RF. The CS cost was most robust in terms of both NNI and SPR edit operations.

## 4. CONCLUSION

A key objective in phylogenetics is to construct a plausible hypothesis of the evolutionary history of an organism. Merging different datasets and gene trees into larger species trees and supertrees can improve the resolution of evolutionary inference. To achieve this, it is necessary to develop methods that can estimate uncertainty and identify conflicts in different gene trees and datasets. Our study introduces an asymmetric CA cost, which is based on determining the minimum cost for a cluster in the source tree for every cluster in the target tree. We have also developed efficient algorithms for calculating the CA cost and determining its diameter. To measure the asymmetry of CA cost, we define a separation diameter as the maximum difference between the two directions that CA cost can be computed. Furthermore, we present a more interpretable cost definition in which cluster differences are normalized by their respective cluster sizes. We have also derived a theoretical diameter for the CS cost and experimentally demonstrated its robustness to tree error.

A promising implication of developing this measure is its future use in the inference of phylogenetic networks (Huson and Bryant, 2006). Some approaches to phylogenetic network inference use tree distance measures to analyze a collection of input trees to seek a minimum reticulation network with the smallest number of reticulation vertices into which the input trees can be embedded (e.g., Markin et al., 2019). The use of RF distance in clustering algorithms often leads to many shallow reticulation events, as the algorithm tries to match clusters fully. To overcome this, we suggest applying CA/CS costs in phylogenetic network inference. This approach allows minor cluster mismatches in smaller clusters to be overlooked, enabling the algorithm to focus on identifying significant reticulation events higher up on the phylogeny (Kong et al., 2022).

The software for computing and visualizing the CA cost is being prepared for release and available upon request from the authors.
